# Molecular identification of slow rusting resistance *Lr46/Yr29* gene locus in selected triticale (*× Triticosecale* Wittmack) cultivars

**DOI:** 10.1007/s13353-020-00562-8

**Published:** 2020-05-18

**Authors:** Roksana Skowrońska, Agnieszka Tomkowiak, Jerzy Nawracała, Michał T. Kwiatek

**Affiliations:** Department of Genetics and Plant Breeding, Faculty of Agronomy and Bioengineering, Poznań University of Life Sciences, 11 Dojazd Str, 60-632 Poznań, Poland

**Keywords:** *csLV46G22*, Molecular markers, *Lr46*, Leaf tip necrosis, Slow rusting, Triticale, *Xwmc44*

## Abstract

Recently, leaf rust and yellow rust caused by the fungi *Puccinia triticina* Erikss. and *P. striiformis* Westend f. sp. *tritici* Eriks and Henn are diseases of increasing threat in triticale (*× Triticosecale* Wittmack, AABBRR, 2*n* = 6x = 42) growing areas. The use of genetic resistance is considered the most economical, effective and environmentally friendly method to control the disease and minimize the use of fungicides. Currently, breeding programs mainly relied on race-specific *Lr* and *Yr* genes (*R*), but new races of the rust fungi frequently defeat resistance. There is a small group of genes that causes partial type of resistance (*PR*) that are characterized by a slow epidemic build up despite a high infection type. In wheat slow rusting resistance genes displayed longer latent periods, low infection frequencies, smaller pustule size and less spore production. Slow rusting *Lr46/Yr29* gene*,* located on chromosome 1B, is being exploited in many wheat breeding programs. So far, there is no information about slow rusting genes in triticale. This paper showed significant differences between the results of identification of wheat molecular markers *Xwmc44* and *csLV46G22* associated with *Lr46/Yr29* in twenty triticale cultivars, which were characterized by high levels of field resistance to leaf and yellow rust. The *csLV46G22res* marker has been identified in the following cultivars: Kasyno, Mamut and Puzon. Belcanto and Kasyno showed the highest resistance levels in three-year (2016–2018), leaf and yellow rust severity tests under post-registration variety testing program (PDO)*.* Leaf tip necrosis, a phenotypic trait associated with *Lr34/Yr18* and *Lr46/Yr29* was observed, among others, to Belcanto and Kasyno, which showed the highest resistance for leaf rust and yellow rust. Kasyno could be considered to have *Lr46/Yr29* and can be used as a source of slow rust resistance in breeding and importantly as a component of gene pyramiding in triticale.

## Introduction

Triticale (× *Triticosecale* Wittmack, 2*n* = 6x = 42, AABBRR genomes) is a man-made amphiploid hybrid produced from the crossing of female parent hexaploid or tetraploid wheat (*Triticum* sp.) and male parent rye (*Secale cereale* L.) (Ammar et al. 2004). It is mostly used in animal feed and biofuel production (Feuillet et al. 2008; McGoverin et al. 2011; Martinek et al. 2008). Triticale, since its commercialization, has shown good resistance to a wide spectrum of pathogens, especially to rusts (Mergoum et al. 2004). As the triticale area harvested has increased, new races of pathogens have adapted to this host (Oettler 2005) and have led to the rapid erosion of effective resistance genes. Leaf rust is one of the most important diseases of wheat (Kolmer 2005), but the pathogen has also been reported on triticale crops (Sodekiewicz and Strzembicka 2004). Leaf rust on triticale is caused by pathotypes of the wheat leaf rust pathogen *Puccinia triticina* that have become virulent to triticale genotypes (Sodekiewicz et al. 2008; Mikhailova et al. 2009). Triticale is annually infected by the same spectrum of pathogens as its parents—wheat and rye (Audenaert et al. 2014). To minimize the use of plant protection products, it is necessary to search for and introduce new sources of resistance to varieties. The genetic origin of leaf rust resistance genes in triticale has been studied by several authors. Singh and McIntosh (1990) showed that leaf rust resistance in five triticale varieties was controlled by a single gene designated *LrSatu*. Wilson and Shaner (1989) studied the inheritance of resistance to culture 7434–1-1 T of *Puccinia recondita* f. sp. *tritici* in four triticales that were selected as potential sources of resistance genes for wheat and described genes for hypersensitive resistance and slow rusting genes in triticale. In Poland, Grzesik and Strzembicka (2003) analyzed leaf rust resistance in three triticale cultivars and showed that the resistance of these cultivars was controlled by the hypersensitive resistance genes described by Wilson and Shaner (1989). Singh and Saari (1991) identified four resistance genes in three genotypes and two additional genes in triticale in Mexico. Stuchlíková and Bartos (1980) analyzed the genetics of resistance to leaf rust in five varieties of triticale in F_2_ and F_3_ and postulated five different genes for resistance to leaf rust. Mikhailova et al. (2009) tested 416 triticale from the Vavilov All-Russian Research Institute of Plant Industry and identified 17 leaf rust resistant cultivar. Hanzalová and Bartoš (2011) studied resistance of triticale to wheat leaf rust and analyzed whether specific differences in virulence exist between wheat leaf rust isolates attacking wheat and isolates attacking triticale. They found that leaf rust isolates from triticale were virulent to a higher number of triticale cultivars than isolates collected from wheat.

One of the most effective and environmentally sound method to control disease is breeding resistant varieties (Dinh et al. 2020). To date, more than 80 genes and alleles of leaf rust resistance (*Lr*) have been identified and described (Mcintosh et al. 2017), but most of this genes are race-specific for hypersensitive resistance (HR). HR genes are very effective in reducing the epidemic build up and easy to introduce in breeding programs because of their monogenic nature, but the resistance provided by these genes can be short-lived as new races of pathogen continue to evolve (Martinez et al. 2001). Kroupin et al. (2019) analyzed the collection of spring triticale accessions for the presence of genes *Lr9*, *Lr12*, *Lr19*, *Lr24*, *Lr25*, *Lr28*, *Lr29* and *Lr47* with the use of molecular markers and isogenic lines carrying target genes. They showed that the gene pool of spring triticale is extremely depleted in leaf rust resistance genes and therefore necessitating work on the introgression of new resistance genes both from the known donor lines of triticale and from bread wheat. It is necessary to search for new sources of resistance or improving intrinsically resistance by gene pyramiding or by use of multilines (McCallum et al. 2007). Another possibility is to incorporate genes that provide partial type of resistance (PR), also known as slow rusting genes or adult plant resistance (APR). PR is a polygenic trait (Parlevliet 1979; Qi et al. 1998) characterized by slow disease progress in the field despite a compatible host reaction (Caldwell 1968). Adult plant resistance (APR) have historically been more durable than race-specific genes (Boyd 2006; Krattinger et al. 2009; Lowe et al. 2011). Slow rusting resistance genes have small to intermediate effects when present alone, so a higher level of resistance is obtained by combining several genes (Singh et al. 2000). In wheat at least seven leaf rust resistance genes are known as slow rusting genes: *Lr34/Yr18* (Singh 1992), *Lr46/Yr29* (Singh et al. 1998), *Lr67/Yr46* (Dyck and Samborski 1979), *Lr68* (Herrera-Foessel et al. 2012), *Lr75* (Singla et al. 2017), *Lr77* (Kolmer et al. 2018a) and *Lr78* (Kolmer et al. 2018b). *Lr34/Yr18* is the leaf rust APR gene with the longest history of resistance, because it has remained effective for almost 100 years (Ellis et al. 2014). *Lr34/Yr18* is the best known slow rusting gene so far. It encodes a modified ATP-binding cassette transporter (Krattinger et al. 2009), and it has been reported to cause an increase in latency period, in percentage of early aborted colonies not associated with cell necrosis and a decrease of colony size (Rubiales and Niks 1995).

*Lr46/Yr29* is the second named gene involved in slow rusting resistance to leaf rust in wheat. *Lr46/Yr29* has provided partial APR to leaf and stripe rust for more than 60 years (Kolmer et al. 2015). It was first described in cultivar Pavon 76 and located on long arm of chromosome 1B (Singh et al. 1998). The effect of *Lr46/Yr29* is similar, but smaller than that of *Lr34/Yr18* in adult plants (Martinez et al. 2001). Lagudah (2011) indicated that *Lr46/Yr29* is more effective in cooler environments and the presence of other *Lr* genes may influence expression of *Lr46*/*Yr29*. William et al. (2003) found that *Lr46* is linked or pleiotropic to *Yr29* stripe rust resistance gene*.* Similarly, the close linkage of *Lr34* slow rusting gene to *Yr18* stripe rust resistance gene was identified as well. Wheat genotypes with gene *Lr46/Yr29* were also determined to have stem rust *Sr58* resistance gene (Singh et al. 2013) and powdery mildew (*Pm39*) resistance gene (Lillemo et al. 2008).

**Fig. 1:**
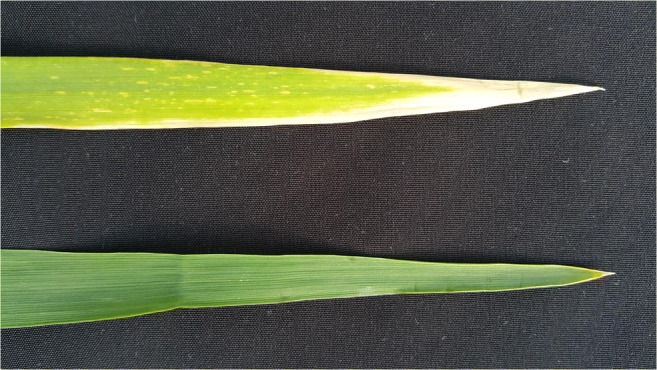
Symptoms of leaf tip necrosis on the flag leaf of Kasyno (up) compared with flag leaf of Grenado (down)

Both genes (*Lr34/Yr18* and *Lr46/Yr29*) are associated with a specific phenotypic trait, leaf tip necrosis (LTN) (Singh 1992; Rosewarne et al. 2006). The symptoms could be described as a dying back of the flag leaf from the tip of the leaf (Fig. 1). Leaf tip necrosis is observed to some extent in all wheat varieties containing the leaf rust gene resistance gene *Lr34*. The LTN trait was described by Singh (1992) to be associated with *Lr34/Yr18* locus by investigating a number of crosses between *Lr34/Yr18*/LTN positive lines and *Lr34/Yr18*/LTN negative lines. It was confirmed by Schnurbusch et al. (2004) in winter bread wheat cv. “Forno,” which has a *Lr34* locus associated with LTN. Rosewarne et al. (2006) used field assays to score for both leaf and yellow rust in an Avocet-*YrA* × Attila population that segregates for several slow rusting leaf and yellow rust resistance genes. What is interesting, the offspring population segregated for LTN, which was interpreted as pleiotropic or closely linked to the *Lr46/Yr29* locus, and *Ltn2* gene was suggested designation to this locus (Rosewarne et al. 2006).

So far, there is no information about slow rusting genes in triticale. Wheat cultivars with slow rusting resistance genes displayed longer latent periods, low infection frequencies, smaller pustule size and less spore production. Considering the location of slow rust genes on wheat chromosomes, there is a presumption that it is possible to identify the *Lr46/Yr29*, *Lr68*, *Lr75* and *Lr77* genes (located on 1BL; 7BL; 1BS and 3BL chromosomes, respectively) in the triticale varieties. Other slow rusting genes—*Lr34*/*Yr18*, *Lr67*/*Yr46* and *Lr78* are located on the D genome (7DS, 4DL and 5DS, respectively), which is not present in the triticale genotype. It is entirely possible, that wheat donors of *Lr46/Yr29* gene were used in triticale breeding programs. The cross of the hexaploid triticale with and hexaploid bread wheat carrying *Lr46* (e.g., Pavon 76), followed by further backcross to triticale could explain its presence in triticale cultivars. It is reported that various wheat-rye translocation lines have been developed to increase genetic variation in wheat and triticale genomes, especially to transfer disease resistance genes and to improve grain yield (Kwiatek and Nawracała 2018). For example, Pavon 1RS near isogenic lines, such as “Pavon 76,” “Pavon 1RS_(*K*)_.1AL,” “Pavon 1RS_(*K*)_.1BL” and “Pavon 1RS_(*K*)_.1DL” developed by Lukaszewski (1993, 2000) are widely used in breeding programs of wheat and triticale (Waines and Ehdaie 2007). The second alternative is the presence of *Lr46* in the tetraploid parent used to develop primary triticale (Herrera-Foessel et al. 2012, Li et al. 2020).

Several molecular markers have been developed for *Lr46/Yr29* identification. At first, this locus was mapped on the long arm of 1B chromosome using AFLP markers (Wilson and Shaner 1989). Suenaga et al. (2003) revealed that the microsatellite locus *Xwmc44* is located 5.6 cm proximal to the putative QTL for *Lr46/Yr29*. Moreover, it is reported that *Lr46/Yr29* locus was mapped distal to *Xwmc44*, approximately 5–15 cm, and proximal to *Xgwm259*, approximately 20 cm (https://maswheat.ucdavis.edu/protocols/Lr46). Microsatellite locus *Xbarc80* is located 10–11 cm distal to *Xgwm259* and is recommended as an alternative distal marker (Lowe et al. 2011). Lagudah et al. (2009, personal communication) developed a cleaved amplified polymorphic sequence (CAPS) *csLV46G22* marker which is reported as the closest marker for the *Lr46/Yr29* gene, so far (Cobo et al. 2019, Lillemo et al. 2008, Ren et al. 2017). Among all markers available, two closest markers (*Xwmc44* and *csLV46G22*) linked to *Lr46/Yr29* were chosen in this study to postulate the presence of *Lr46/Yr29* gene in fourteen winter and six spring Polish cultivars of triticale.

## Materials and methods

This study was performed on twenty Polish triticale cultivars derived from Danko Hodowla Roślin Sp. z o.o. breeding company (Table 1). Bread wheat (*Triticum aestivum* L). cv. “Pavon F76” (PI 520003) derived from the National Small Grains Collection, the Agriculture Research Station in Aberdeen, was the reference material.

**Table 1:** Presence of *Lr46/Yr29* gene in tested triticale winter varieties

No.	Cultivar	*Molecular markers*		Leaf tip necrosis (LTN)	Leaf rust resistance (scale 1–9)*	Yellow rust resistance (scale 1–9)*
		*Xwmc44*	*csLV46G22*			
1.	Avocado	–	–	–	8.3	8.2
2.	Belcanto	–	–	+	8.6	8.7
3.	Dolindo	+	–	+	8.5	8.0
4.	Fredro	+	–	–	7.5	7.5
5.	Kasyno	–	+	+	8.5	8.8
6.	Maestozo	–	–	–	8.3	7.3
7.	Orinoko	+	–	–	8.2	7.8
8.	Pizarro	+	–	+	8.0	8.4
9.	Porto	+	–	+	8.5	8.3
10.	Rotondo	–	–	–	8.0	6.5
11.	Subito	–	–	–	8.1	7.5
12.	Trapero	–	–	–	8.3	8.4
13.	Trismart	+	–	–	6.8	6.8
14.	Twingo	–	–	–	8.0	8.3
Total resistance (scale 1–9)* mean					*8*.*1*	*7*.*9*
Resistance (scale 1–9)* mean for Xwmc44res					*7*.*9*	*7*.*8*
Resistance (scale 1–9)* mean for Xwmc44sus					*8*.*3*	*8*.*0*
Resistance (scale 1–9)* mean for csLV46G22res					*8*.*5*	*8*.*8*
Resistance (scale 1–9)* mean for csLV46G22sus					*8*.*1*	*7*.*8*
Resistance (scale 1–9)* mean for LTN+					*8*.*4*	*8*.*4*
Resistance (scale 1–9)* mean for LTN−					*7*.*9*	*7*.*6*

*Scale of the Research Centre for Cultivar Testing (COBORU) in Słupia Wielka (Poland). 9—most resistant; 1—most susceptible. Mean data collected by post-registration variety testing (PDO) in 2016–2018 (Drażkiewicz 2019)

DNA was isolated from the leaves of 10-day-old seedlings with the use of GeneMATRIX Plant and Fungi DNA Purification Kit (EURx Ltd., Poland). DNA concentration and quality was determined using the DeNovix spectrophotometer (DeNovix Inc., USA). The samples were diluted with Tris buffer (EURx Ltd., Poland) to obtain a uniform concentration of 50 ng/μL. To identify the *Lr46/Yr29* gene, the molecular markers *Xwmc44* and *csLV46G22* was used. The sequences of primers are as follows: Xwmc44F 5′-GGT CTT CTG GGC TTT GAT CCT G-3′ and Xwmc44R 5′-GTT GCT AGG GAC CCG TAG TGG-3′. The CAPS marker *csLV46G22* tightly linked to *Lr46/Yr29* was kindly provided by Dr. Evans Lagudah, CSIRO Plant Industry, Canberra, Australia (personal communication, 2020). The 25 μL polymerase chain reaction (PCR) mixture for *Xwmc44* and *csLV46G22* consisted of the following: 12.5 μL 2x PCR TaqNovaHs PCR Master Mix (Blirt, Poland), which included 2× concentrated PCR reaction buffer, 4 mm MgCl2; 1.6 mm dNTPs mix (0.4 mm of each dNTP); 1 μL Xwmc44 forward primer; 1 μL Xwmc44 reverse primer (the concentration for each primer was 100 μM); 2 μL DNA template; and 6.5 μL PCR grade water. The PCR was modified on the basis of a standard protocol. The primer annealing temperature of the marker primers was 61 °C for *Xwmc44* (Suenaga et al. 2003). The final PCR reaction consisted of initial denaturation at 94 °C for 5 min, followed by 35 cycles (denaturation, 94 °C for 45 s; primer annealing, 61 or 64 °C for 30 s; elongation, 72 °C for 1 min), followed by the final extension for 7 min at 72 °C and storage at 4 °C. The *csLV46G22* PCR amplification products were digested with the restriction enzyme BspEI in thermocycler at 37 °C for 1 h (Lagudah, pers. comm. 2020; Ponce-Molina et al. 2018). PCR and digestion was carried out using the Labcycler thermal cyclers (SensoQuest, Germany). The products of amplification were prepared by adding 0.5 Midori Green Direct (Nippon Genetics Europe, Germany) to each tube. The products were separated for one and a half hour using 2% agarose (Sigma-Aldrich, Poland) gel in 1× TBE buffer (BioShop, Canada) at 100 V. To visualize the PCR products, a Molecular Imager Gel Doc™ XR UV system was used with the Biorad Bio Image™ Software (Biorad, USA).

The mean scores of leaf and yellow rust severity were adapted from post-registration variety testing program (PDO) for winter (Drażkiewicz 2019) and spring triticale cultivars (Najewski 2019). This program was performed by the Research Centre for Cultivar Testing (COBORU) and included 3 years (2016–2018) of field scoring of natural infection of *P. triticina* and *P. stiiformis* in fifty localizations in Poland (Zych 2019).

Leaf tip necrosis was scored for twenty triticale accessions in field trial at the Poznan University of Life Sciences. Ten randomly chosen flag leaves were observed and evaluated using positive/negative (LTN+/LTN−) scores (Fig. 1).

## Results and discussion

Most leaf rust resistance genes are race specific (R) and effective during all of the host life cycle, being called seedling genes. Seedling resistance is usually manifested by hypersensitive resistance response (Bolton et al. 2008). Leaf rust resistance conditioned by adult plant genes (APR) can be expressed only at adult plant stage. Some adult plant resistance genes are characterized by conferring partial resistance, which is associated with a slow rusting development instead of a rapid hypersensitive response. Slow rusting genes result in fewer and smaller uredinia and longer latent periods (Lagudah et al. 2009). The partial resistance genes condition longstanding effectiveness. *Lr46* is located on long arm of 1B chromosome and confers a comparable non-hypersensitive type of defense to infection of *P. triticina* as *Lr34* (7DS), but its effect is smaller than that of *Lr34* (Martinez et al. 2001).

**Fig. 2:**
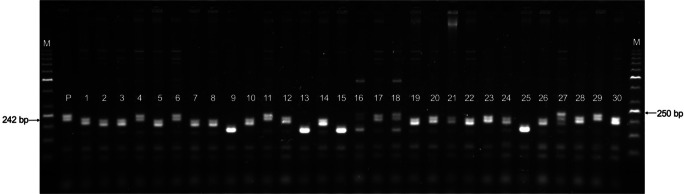
Electropherogram showing the presence of *Xwmc44* marker in the triticale varieties. *M*, GeneRuler 50 bp DNA ladder (Nippon Genetic Europe, Germany); *P*, Pavon F76; 1–30, triticale varieties

In this study, we assumed that *Lr46* gene located on 1B chromosomes could be present in some of triticale cultivars, considering different breeding pathways of primary and secondary triticale. We screened twenty Polish triticale cultivars for *Lr46* using two closest molecular markers and showed that the microsatellite marker *Xwmc44* do not line up with the CAPS marker *csLV46G22* analyses. Microsatellite locus of *Xwmc44* marker is located 5.6 cm proximal to the putative QTL for *Lr46/Yr19* (Suenaga et al. 2003). For comparison, recent maps for *Lr46/Yr19* from Pavon 76 place this locus between *TraesCS1B01G453900* and *csLV46G22* (Lagudah, personal comm.). *Xwmc44* marker was identified in 6 winter cultivars: Dolindo, Fredro, Orinoko, Pizarro, Porto and Trismart (Fig. 2, Tables 1 and 2). Results for *Xwmc44* resistance allele do not coincided with the CAPS marker *csLV46G22res* tightly linked to *Lr46/Yr19*. A specific product of *csLV46G22* marker was observed in three other triticale cultivars: Kasyno (winter cultivar), Mamut and Puzon (spring cultivars) (Tables 1 and 2). Considering the durability of *Lr46* expression, the results of molecular marker analysis were compared with leaf and yellow rust severity in these triticales tested in the field under post-registration variety testing program (PDO 2016–2018; Drażkiewicz 2019, Najewski 2019). Belcanto and Kasyno were the most resistant for infections of both *P. titicina* and *P. stiiformis* causing leaf and yellow rust, respectively. The mean score for leaf rust resistance ranged between 6.8 and 8.6 for winter cultivars (Table 1) and 7.5–8.4 for spring cultivars screened in this study (Table 1). Simultaneously, the mean score for yellow rust resistance ranged between 6.5 and 8.8 for winter cultivars (Table 1) and 8.1–8.7 for spring cultivars (Table 2). What is interesting is that the mean scores for leaf and yellow rust resistance for winter cultivars carrying *Xwmm44res* allele were lower than the mean scores for *Xwmc44sus* cultivars (Table 1). Considering the second marker, the mean scores for leaf and yellow rust resistance for *csLV46G22res* cultivars were higher than mean scores for *csLV46G22sus* cultivars (Tables 1 and 2) with one exception. The scores of yellow rust severity were comparable comparing spring cultivars carrying *csLV46G22res* and *csLV46G22sus* (Table 2). The results were also compared with leaf tip necrosis (LTN) analysis. This trait is associated with the *Lr34 and Lr46* genes and is a useful phenotypic marker to identify the presence of those genes in wheat lines (Rosewarne et al. 2006). LTN trait was observed only in five winter cultivars (Belcanto, Dolindo, Kasyno, Pizzarro and Porto). The leaf and yellow resistance scores for LTN+ cultivars were higher comparing to LTN− cultivars. Among LTN+ winter cultivars, Dolindo, Pizzaro and Porto carried *Xwmc44res* allele, when Kasyno was identified to have *csLV46G22res* allele.

**Table 2:** Presence of *Lr46/Yr29* gene in tested triticale spring varieties

No.	Cultivar	*Molecular markers*		Leaf tip necrosis (LTN)	Leaf rust resistance (scale 1–9)*	Yellow rust resistance (scale 1–9)*
		*Xwmc44*	*csLV46G22*			
1.	Dublet	–	–	–	7.5	8.5
2.	Mamut	–	+	–	8.3	8.7
3.	Mazur	–	–	–	8.2	8.5
4.	Puzon	–	+	–	8.3	8.1
5.	Santos	–	–	–	8.4	8.5
6.	Sopot	–	–	–	8.0	8.4
Total resistance (scale 1–9)* mean					*8*.*1*	*8*.*5*
Resistance (scale 1–9)* mean for Xwmc44res					*n/a*	*n/a*
Resistance (scale 1–9)* mean for Xwmc44sus					*8*.*1*	*8*.*5*
Resistance (scale 1–9)* mean for csLV46G22res					*8*.*3*	*8*.*4*
Resistance (scale 1–9)* mean for csLV46G22sus					*8.0*	*8*.*5*
Resistance (scale 1–9)* mean for LTN+					*n/a*	*n/a*
Resistance (scale 1–9)* mean for LTN−					*8*.*1*	*8*.*5*

*Scale of the Research Centre for Cultivar Testing (COBORU) in Słupia Wielka (Poland). 9, most resistant; 1, most susceptible. Mean data collected by post-registration variety testing (PDO) in 2016–2018 (Najewski 2019)

Considering the results of molecular markers compared with 3 years of leaf and yellow rust severity tests and LTN trait scoring, it could be assumed that triticale cv. Kasyno could be considered to have *Lr46/Yr29* gene. What is more basing on high levels of leaf and yellow rust resistance and the presence of LTN trait is that triticale cv. Belcanto could be suspected to have *Lr34/Yr18* gene. However, this requires additional molecular marker investigation, which falls outside the scope of this study. In addition, these cultivars may serve as the starting material for pyramiding slow rusting resistance genes in triticale genotypes.

## References

[CR1] Ammar K, Mergoum M, Rajaram S, Mergoun M, Gomez-Macpherson H (2004). The history and evolution of triticale. Triticale improvement and production.

[CR2] Audenaert K, Troch V, Landschoot S, Haesaert G (2014). Biotic stresses in the anthropogenic hybrid triticale (× *Triticosecale* Wittmack): current knowledge and breeding challenges. Eur J Plant Pathol.

[CR3] Bolton MD, Kolmer JA, Garvin DF (2008). Wheat leaf rust caused by *Puccinia triticina*. Mol Plant Pathol.

[CR4] Boyd LA (2006). Can the durability of resistance be predicted?. J Sci Food Agric.

[CR5] Caldwell RM (1968). Breeding for general and/or specific plant disease resistance. Proceedings of the third international wheat genetics symposium, Canberra, Australia.

[CR6] Cobo N, Wanjugi H, Lagudah E, Dubcovsky J (2019). A high-resolution map of wheat QYr.Ucw-1BL, an adult plant stripe rust resistance locus in the same chromosomal region as Yr29. Plant Genome.

[CR7] Dinh HX, Singh D, Periyannan S, Park RF, Pourkheirandish M (2020) Molecular genetics of leaf rust resistance in wheat and barley. Theor Appl Genet. 10.1007/s00122-020-03570-8 10.1007/s00122-020-03570-8 32128617

[CR8] Drażkiewicz K. (2019) Pszenżyto. Pszenżyto ozime. [in:] Lista opisowa odmian roślin rolniczych 2019. http://www.coboru.pl/Publikacje_COBORU/Listy_opisowe/LOORR%20-%20zbozowe%202019.pdf. Accessed 4 May 2020

[CR9] Dyck PL, Samborski DJ (1979). Adult-plant leaf rust resistance in PI 250413, an introduction of common wheat. Can J Plant Sci.

[CR10] Ellis JG, Lagudah ES, Spielmeyer W, Dodds PN (2014). The past, present and future of breeding rust resistant wheat. Front Plant Sci.

[CR11] Feuillet C, Langridge P, Waugh R (2008). Cereal breeding takes a walk on the wild side. Trends Genet.

[CR12] Grzesik H, Strzembicka A (2003). Resistance of some winter triticale varieties to leaf rust [*Puccinia recondita* f.sp. *tritici*]. Biul Inst Hod i Aklim Roślin.

[CR13] Hanzalová A, Bartoš P (2011). Resistance of triticale to wheat leaf rust (*Puccinia triticina*). Czech J Genet Plant Breed.

[CR14] Herrera-Foessel SA, Singh RP, Huerta-Espino J, Rosewarne GM, Periyannan SK, Viccars L, Calvo-Salazar V, Lan C, Lagudah ES (2012). *Lr68*: a new gene conferring slow rusting resistance to leaf rust in wheat. Theor Appl Genet.

[CR15] Kolmer JA (2005). Tracking wheat rust on a continental scale. Curr Opin Plant Biol.

[CR16] Kolmer JA, Lagudah ES, Lillemo M, Lin M, Bai G (2015). The *Lr46* gene conditions partial adult-plant resistance to stripe rust, stem rust, and powdery mildew in Thatcher wheat. Crop Sci.

[CR17] Kolmer JA, Su Z, Bernardo A, Bai G, Chao S (2018). Mapping and characterization of the new adult plant leaf rust resistance gene *Lr77* derived from Santa Fe winter wheat. Theor Appl Genet.

[CR18] Kolmer JA, Bernardo A, Bai G, Hayden MJ, Chao S (2018). Adult plant leaf rust resistance derived from Toropi wheat is conditioned by *Lr78* and three minor QTL. Phytopathology.

[CR19] Krattinger SG, Lagudah ES, Spielmeyer W, Singh RP, Huerta-Espino J, McFadden H (2009). A putative ABC transporter confers durable resistance to multiple fungal pathogens in wheat. Science.

[CR20] Kroupin PY, Gruzdev IV, Divashuk MG, Bazhenov MS, Kocheshkova AA, Chernook AG (2019). Analysis of spring triticale collection for leaf rust resistance genes with PCR markers. Russ J Genet.

[CR21] Kwiatek MT, Nawracała J (2018). Chromosome manipulations for progres of triticale (×*Triticosecale* Wittmack) breeding. Plant Breed.

[CR22] Lagudah ES (2011). Molecular genetics of race non-specific rust resistance in wheat. Euphytica.

[CR23] Lagudah ES, Krattinger SG, Herrera-Foessel S, Singh RP, Huerta-Espino J, Spielmeyer W, Keller B (2009). Gene-specific markers for the wheat gene *Lr34/Yr18/Pm38* which confers resistance to multiple fungal pathogens. Theor Appl Genet.

[CR24] Li C, Lu X, Zhang Y, Liu N, Li C, Zheng W (2020). The complete mitochondrial genomes of *Puccinia striiformis* f. sp. *tritici* and *Puccinia recondita* f. sp. *tritici*. Mitochondrial DNA Part B.

[CR25] Lillemo M, Asalf B, Singh RP, Huerta-Espino J, Chen XM, He ZH (2008). The adult plant rust resistance loci *Lr34/Yr18* and *Lr46/Yr29* are important determinants of partial resistance to powdery mildew in bread wheat line. Saar Theor Appl Genet.

[CR26] Lowe I, Jankuloski L, Chao S, Chen X, See D, Dubcovsky J (2011). Mapping and validation of QTL which confer partial resistance to broadly virulent post-2000 North American races of stripe rust in hexaploid wheat. Theor Appl Genet.

[CR27] Lukaszewski AJ (1993). Reconstruction in wheat of complete chromosome1B and 1R from 1RS.1BL translocation of Kavkaz origin. Genome.

[CR28] Lukaszewski AJ (2000). Manipulation of the 1RS.1BL translocation inwheat by induced homoeologous recombination. Crop Sci.

[CR29] Martinek P, Vinterová M, Burešová I, Vyhnánek T (2008). Agronomic and quality characteristics of triticale (×*Triticosecale* Wittmack) with HMW glutenin subunits 5+10. J Cereal Sci.

[CR30] Martinez F, Niks RE, Singh RP, Rubiales D (2001). Characterization of *Lr46*, a gene conferring partial resistance to wheat leaf rust. Hereditas.

[CR31] McCallum BD, Fetc T, Chong J (2007). Cereal rust control in Canada. Aust J Agric Res.

[CR32] McGoverin CM, Snyders F, Muller N, Botes W, Fox G, Manley M (2011). A review of triticale uses and the effect of growth environment on grain quality. J Sci Food Agric.

[CR33] McIntosh RA, Dubcovsky J, Rogers WJ, Morris C, Xia XC (2017) Catalogue of gene symbols for wheat: 2017 supplement. https://shigen.nig.ac.jp/wheat/komugi/genes/macgene/supplement2017.pdf. Accessed 4 May 2020

[CR34] Mergoum M, Pfeiffer WH, Pena RJ, Ammar K, Rajaram S, Mergoum M, Gomez-Maxpherson H (2004). Triticale crop improvement: the CIMMYT programme. Triticale improvement and production. FAO plant production and protection paper 179.

[CR35] Mikhailova L, Merezhko AF, Funtikova EY (2009). Triticale diversity in leaf rust resistance. Russ Agric Sci.

[CR36] Najewski (2019) Pszenżyto. Pszenżyto jare. [in:] Lista opisowa odmian roślin rolniczych 2019. http://www.coboru.pl/Publikacje_COBORU/Listy_opisowe/LOORR%20-%20zbozowe%202019.pdf. Accessed 4 May 2020

[CR37] Oettler G (2005). The fortune of a botanical curiosity – triticale: past, present and future. J Agric Sci.

[CR38] Parlevliet JE (1979). Further evidences of polygenic inheri- tance of partial resistance in barley to leaf rust, *Puccinia hordei*. Euphytica.

[CR39] Ponce-Molina LJ, Huerta-Espino J, Singh RP, Basnet BR, Lagudah E, Aguilar-Rincón VH (2018). Characterization of adult plant resistance to leaf rust and stripe rust in Indian wheat cultivar ‘New Pusa 876’. Crop Sci.

[CR40] Qi X, Niks RE, Stam P, Lindhout P (1998). Identification of QTLs for partial resistance to leaf rust (*Puccinia hordei*) in barley. Theor Appl Genet.

[CR41] Ren Y, Singh RP, Basnet BR, Lan CX, Huerta-Espino J, Lagudah ES (2017). Identification and mapping of adult plant resistance loci to leaf rust and stripe rust in common wheat cultivar Kundan. Plant Dis.

[CR42] Rosewarne GM, Singh RP, Huerta-Espino J, William HM, Bouchet S, Cloutier S, Lagudah ES (2006). Leaf tip necrosis, molecular markers and β1-proteasome subunits associated with the slow rusting resistance genes *Lr46/Yr29*. Theor Appl Genet.

[CR43] Rubiales D, Niks RE (1995). Characterization of *Lr34*, a major gene conferring nonhypersensitive resistance to wheat leaf rust. Plant Dis.

[CR44] Schnurbusch T, Bossolini E, Messmer M, Keller B (2004). Tagging and validation of a major quantitative trait locus for leaf rust resistance and leaf tip necrosis in winter wheat cultivar Forno. Phytopathology.

[CR45] Singh RP (1992). Expression of wheat leaf rust resistance gene *Lr34* in seedlings and adult plants. Plant Dis.

[CR46] Singh SJ, McIntosh RA (1990). Linkage and expression of genes for resistance to leaf rust and stem rust in triticale. Genome.

[CR47] Singh RP, Saari EE (1991) Biotic stresses in triticale. In: 2. Proceedings of the International Triticale Symposium. Passo Fundo (Brazil). 1–5 Oct 1990

[CR48] Singh RP, Mujeeb-Kazi A, Huerta-Espino J (1998). *Lr46/Yr19*: a gene conferring slow-rusting resistance to leaf rust in wheat. Phytopatholog.

[CR49] Singh RP, Huerta-Espino J, Rajaram S (2000). Achieving near-immunity to leaf and stripe rusts in wheat by combining slow rusting resistance genes. Acta Phytopathol Entomol Hunga.

[CR50] Singh RP, Herrera-Foessel SA, Huerta-Espino J, Lan CX, Basnet BR, Bhavani S et al (2013) Pleiotropic gene *Lr46/Yr29/Pm39/Ltn2* confers slow rusting, adult plant resistance to wheat stem rust fungus. In: proceedings of the Borlaug global rust initiative technical workshop, 19–22 Aug. 2013, New Delhi, India. Indian Council of Agricultural Research, New Delhi. p. 17.1

[CR51] Singla J, Lüthi L, Wicker T, Bansal U, Krattinger SG, Keller B (2017). Characterization of *Lr75*: a partial, broad-spectrum leaf rust resistance gene in wheat. Theor Appl Genet.

[CR52] Sodekiewicz W, Strzembicka A (2004). Application of *Triticum monococcum* for the improvement of triticale resistance to leaf rust (*Puccinia triticina*). Plant Breed.

[CR53] Sodekiewicz W, Strzembicka A, Apolinarska B (2008). Chromosomal location in triticale of leaf rust resistance genes introduced from *Triticum monococcum*. Plant Breed.

[CR54] Stuchlíková E, Bartos P (1980). Genetic analysis of resistance to brown rust in triticale. Genetika a Slechteni.

[CR55] Suenaga K, Singh RP, Huerta-Espino J, William HM (2003). Microsatellite markers for genes Lr34/Yr18 and other quantitative trait loci for leaf rust and stripe rust resistance in bread wheat. Phytopathology..

[CR56] Waines JG, Ehdaie B (2007). Domestication and crop physiology: roots of green-revolution wheat. Ann Bot.

[CR57] William M, Singh RP, Huerta-Espino J, Islas SO, Hoisington D (2003). Molecular marker mapping of leaf rust resistance gene Lr46 and its association with stripe rust resistance gene Yr29 in wheat. Phytopathology.

[CR58] Wilson J, Shaner G (1989). Inheritance of the leaf rust resistance in four triticale cultivars. Phytopathology.

[CR59] Zych J (2019) Wprowadzenie [in:] Lista opisowa odmian roślin rolniczych 2019. http://www.coboru.pl/Publikacje_COBORU/Listy_opisowe/LOORR%20-%20zbozowe%202019.pdf. Accessed 4 May 2020

